# Fuzhu jiangtang granules combined with metformin reduces insulin resistance in skeletal muscle of diabetic rats via PI3K/Akt signaling

**DOI:** 10.1080/13880209.2019.1659831

**Published:** 2019-09-23

**Authors:** Yunsong Cao, Wen Sun, Guangyuan Xu

**Affiliations:** a Department of Nephrology, Dongfang Hospital, Beijing University of Traditional Chinese Medicine, Beijing, China;; b Key Laboratory of Health Cultivation of the Ministry of Education, Beijing University of Chinese Medicine, Beijing, China;; c Department of Traditional Chinese Medicine, Fuxing Hospital, the Eighth Clinical Medical College, Capital Medical University, Beijing, China

**Keywords:** Antidiabetic drugs, Chinese herbal formula, anti-obesity, antihypoglycemic, antihypolipidemic, antioxidant, insulin signaling pathway

## Abstract

**Context:** Fuzhu Jiangtang Granules (FJG) are a traditional Chinese used in the treatment of diabetes mellitus. However, the antidiabetic mechanism of FJG is not clear.

**Objective:** This study evaluates and determines the antidiabetic mechanism of FJG using a Zucker diabetic fatty (ZDF) rat model.

**Materials and methods:** ZDF (fa/fa) rats were divided into four groups (*n* = 6): diabetes mellitus (DM), metformin (Met, 0.134 g/kg b.w./day), FJG (0.64 g/kg b.w./day), and combination (Com, 0.134 g/kg b.w./day of Met and 0.64 g/kg b.w./day of FJG). Six ZDF (fa/+) rats served as a normal control. After 6 weeks, biochemical parameters gene and protein expression were detected.

**Results:** The FBG, bodyweight, triglyceride (TG), total cholesterol (TC), free fatty acid (FFA), insulin levels, and HOMA-IR were lower in the FJG than in the DM group (*p* < 0.05, *p* < 0.01). In an oral glucose tolerance test, the AUC in the FJG group was significantly lower (*p* < 0.01). The levels of superoxide dismutase and catalase were higher in the FJG than in the DM group (*p* < 0.01); the malondialdehyde content and TNF-α were significantly decreased in the FJG group (*p* < 0.01). FJG increased the mRNA expression of IR and GLUT4 significantly (*p* < 0.05, *p* < 0.01). The protein levels of IR, p-IRS1 tyr989, m-PI3Kp85, p-Akt and GLUT4 were increased in the FJG (*p* < 0.05, *p* < 0.01), but the protein levels of p-IRS1 ser1101/612/307 were significantly decreased in the JG group (*p* < 0.01).

**Discussion and conclusions:** The antidiabetic mechanism of FJG may be related to regulation of the insulin-signaling pathway in skeletal muscle. These aspects require further research.

## Introduction

Diabetes mellitus is a chronic metabolic disorder of multiple etiologies characterized by disturbances in glycemic and lipid metabolism. Currently, impaired insulin secretion and insulin resistance are generally accepted as pathological changes that lead to the development of diabetes. Skeletal muscle is a crucial metabolic organ for insulin-stimulated glucose disposal, and has been implicated in insulin resistance and obesity (Petersen and Shulman 2002; Ceddia 2005). Therefore, it has been extensively studied in relation to type 2 diabetes.

Traditional Chinese medicine (TCM) has been widely used in China for more than 2000 years for the prevention and treatment of diabetes and complications such as diabetic nephropathy (Tong et al. 2012; He et al. 2016; Sun et al. 2016). Fuzhu Jiangtang granules (FJG) consist of many complex ingredients such as *Momordica charantia* L. (Cucurbitaceae), *Polygonatum odoratum* (Mill.) Druce (Asparagaceae), *Morus alba* L. (Moraceae), *Panax notoginseng* (Burk.) F. H. Chen (Araliaceae) and *Cinnamomum cassia* Presl. (Lauraceae). Previous studies have found that Jiang Tang Xiao Ke (JTXK) granules, a Chinese herbal formula similar to FJG, could improve glycemic and lipid metabolism in diabetic rats through its anti-oxidative action and modulation of the phosphoinositide 3-kinase (PI3K)/protein kinase B (Akt) signaling pathway (Zhao et al. 2014; Guo et al. 2016; Zhang et al. 2016; Yu et al. 2017). Despite numerous advances in treatment, there are still many diabetic patients who are unable to achieve adequate glycemic control while on the maximum recommended dose of antidiabetic agents. Thus, attempts should be made to find complementary and alternative therapies to the existing antidiabetic agents to achieve satisfactory outcomes.

Previous studies showed that FJG combined with metformin can improve TC, FFA, FBG and IR in type 2 diabetic rats, and the effect is better than metformin (Guang-Yuan et al. 2017). Therefore, in the present study, we evaluate the effects of FJG on the regulation of glycemic and lipid metabolism, and study the morphological changes of skeletal muscle *in vivo* using a Zucker diabetic fatty (ZDF) rat model. The oral hypoglycemic agent metformin (Met), commonly used to treat diabetes, has been shown to decrease lipid accumulation and is well tolerated by patients (Nyenwe et al. 2011; Viollet et al. 2012). Therefore, Met was selected as the control drug to confirm the effect of FJG on insulin resistance, and was also used to explore the complementary therapeutic effect of FJG. We also investigated the underlying mechanisms by which FJG improves insulin resistance for type 2 diabetic patients.

## Materials and methods

### Preparation of FJG

FJG were prepared and obtained from the Dongfang Hospital, Beijing University of Traditional Chinese Medicine, and verified at Dongfang Hospital (Beijing, China) by Dr. Yun-song Cao. The concentrations of rutin, isoquercitrin, notoginsenoside R1 and ginsenoside Rg1 in the FJG were determined to be 1.6, 2.3, 2.2 and 2.8%, respectively, using high-performance liquid chromatography (HPLC) analysis.

### Experimental animals

Six-week-old male ZDF (fa/fa) rats (*n* = 24, 180–200 g) and age-matched, lean counterparts ZDF (fa/+, *n* = 6, 160–180 g) were obtained from the Beijing Vital River Laboratory Animal Technology Co. Ltd. (license number: SCXK Beijing 2012-0001). The experimental protocol was approved by the ethics committee of the Animal Care and Use Committee of Beijing University of Chinese Medicine. The rats were housed in a specific pathogen-free (SPF) animal laboratory at the institute of basic theory of TCM, the China Academy of Chinese Medical Science (Laboratory animal license: SYXK Beijing 2010-0032). All rats were given access to food and water *ad libitum* and maintained in conditions of 12 h light/dark cycle with standard temperature (23 ± 2 °C) and humidity (55 ± 10%). The ZDF (fa/fa) rats were fed with a high-fat diet (Pumina #5008, PMI®) containing 26.85% protein, 16.71% fat, and 56.44% carbohydrate. The ZDF (fa/+) rats were fed standard chow provided by the Institute of Laboratory Animal Science, Chinese Academy of Medical Sciences.

After maintaining animals on the designated diet for 4 weeks, blood samples were collected from the tail vein to detect baseline plasma glucose levels. A fasting blood glucose (FBG) concentration ≥ 11.0 mM was defined as having diabetes mellitus. The ZDF (fa/fa) rats were divided into four groups (*n* = 6): 1) vehicle-treated diabetic model group (DM), metformin-treated diabetic group (Met, #H20023370; Sino-American Shanghai Squibb Pharmaceutical Ltd., 0.134 g/kg per day), FJG-treated diabetic group (FJG, 0.64 g/kg per day), and combined treatment group with Met and FJG (Combination, 0.134 g/kg per day of Met, 0.64 g/kg per day of FJG). Six ZDF (fa/+) rats served as the normal control group (Nor). An equal volume of normal saline, FJG, or Met was intragastrically administered by gavage to the appropriate rats once daily for 6 weeks. The high-fat diet was maintained throughout the experimental period in all diabetic rats. After the treatment, all rats were anesthetized by intraperitoneal injection of 10% pentobarbital, then blood was collected from abdominal aorta, and the rats died.

### General condition of experimental rats

The general condition of all rats were monitored daily, including mental state, activity, and fur appearance. Body weights were recorded every week throughout the experiment.

### Biochemical analysis

The FBG, triglyceride (TG), total cholesterol (TC) and free fatty acid (FFA) levels were determined by colorimetric assays (SINO-UK Institute of Bio-Tech, Beijing, China). The fasting insulin (FINS) and tumor necrosis factor-alpha (TNF-α) was measured in the serum using enzyme-linked immunosorbent assay (ELISA) kits (SINO-UK Institute of Bio-Tech). Aspartate aminotransferase (AST), serum creatinine (SCr) and blood urea nitrogen (BUN) were measured using an automatic biochemical analyzer (MINDRAY BS-420, China). The levels of superoxide dismutase (SOD), catalase (CAT), and malondialdehyde (MDA) were detected in the serum using an A6 semi-automatic biochemical analyzer (Beijing Shiningsun Tech Co. Ltd., China). The homeostasis model assessment of β-cell function and insulin resistance (HOMA-IR) was calculated with the formula FBG (µIU/mL)×FINS (mmol/L)/22.5.

### Oral glucose tolerance test

At the sixth week, an oral glucose tolerance test (OGTT) was performed on each subject by gastric gavage of a glucose solution (2.0 g/kg bodyweight) following an overnight fasting period. Blood was obtained from the tail vein for glucose determination at 0, 30, 60, and 120 min of the test.

### Histological examination

The skeletal muscle tissue from rats in each group was immersed in 10% neutral buffered formalin for 48 h, embedded in paraffin, and cut into sections (4 µm). Tissue sections were then exposed to dimethylbenzene for dewaxing and an ethanol gradient for rehydration prior to staining with hematoxylin and eosin (H&E, #20140916, Beijing Solarbio Science & Technology Ltd., China). Slides were observed under a light microscope (BX53, Olympus, Japan).

### Real-time quantitative polymerase chain reaction

Total RNA was extracted from frozen rat skeletal muscle tissue using a TRIzol reagent (#98903, Life Technologies, Carlsbad, CA, USA), and total RNA (500 ng) was reverse-transcribed into complementary DNA (cDNA) using the GoScript™ Reverse Transcription System (Promega, Madison, WI, USA). For the quantitative polymerase chain reaction (qPCR), the primer sequences used were as follows: insulin receptor (InsR, 214 bp) forward: 5′-GGCCCGATGCTGAGAACA-3′, reverse 5′-CGTCATTCCAAAGTCTCCGA-3′; glucose transporter type 4 (Glut4, 270 bp) forward: 5′-CGCGGCCTCCTATGAGATAC-3′, reverse 5′-CCTGAGTAGGCGCCAATGA-3′; and glyceraldehyde 3-phosphate dehydrogenase (GAPDH, 146 bp), forward: 5′-GGAAGCTCCGGGAACAAGT-3′, reverse 5′-TGCCAGCCCATGGATTCTC-3′. DNA amplification was performed on an ABI 7500 system (Applied Biosystems, Foster City, CA) with GoTaq® qPCR Master Mix (#0000076581, Promega, Madison, WI, USA) using the following thermal conditions: 95 °C for 30 s and 40 cycles of 95 °C for 5 s and 60 °C for 30 s. Fluorescence intensity was calculated as a ratio of the optical density of the gene of interest to the loading control (GAPDH).

### Western blotting

Antibodies against the InsR, phosphorylated insulin receptor substrate-1 (IRS-1) at ser1101/ser612/ser307, PI3K, PI3Kp85, total Akt, phospho-Akt (p-Akt), Glut4, and β-actin were purchased from Cell Signaling Technology (Danvers, MA, USA). p-IRS-1 (tyr989) was obtained from Santa Cruz Biotechnology (Santa Cruz, CA, USA). Goat anti-rat and rabbit anti-goat secondary antibodies were obtained from Abcam (Cambridge, UK), and goat anti-rabbit secondary antibody was obtained from Cell Signaling Technology.

The frozen rat skeletal muscle tissue was lysed in RIPA Lysis Buffer (#C1053, Applygen Technologies Inc., China). The protein samples were loaded onto polyacrylamide gels and subjected to sodium dodecyl sulfate polyacrylamide gel electrophoresis (SDS-PAGE). Proteins were then transferred to a polyvinylidene difluoride (PVDF) membrane. The membrane was blocked with Blocking One/Blocking One-P (#L4E0085 and #L5G4440, Nacalai Tesque, Kyoto, Japan) at 37 °C for 30 min, followed by incubation with primary antibodies (dilution 1:1000) at 4 °C overnight. After washing, the membrane was incubated with secondary antibodies (dilution 1:10000) at room temperature for 1 h. Bands were detected using an enhanced chemiluminescence (ECL) substrate kit (#170-5060, Bio-Rad Laboratories, Hercules, CA, USA), and the band density was measured quantitatively with an image analysis software (ImageJ 7.0, Research Services Branch, NIH, USA). The values were corrected by reference to the value of β-actin.

### Statistical analysis

The SPSS for Windows version 17.0 software package (SPSS Inc., USA) was used for statistical data analyses. Data were expressed as mean ± standard deviation (SD). The difference between two groups was analyzed by the Student’s *t*-test. One-way analysis of variance (ANOVA) with Bonferroni adjustment was used for multiple comparisons. For the OGTT test, the area under the curve (AUC) was determined and compared using GraphPad Prism version 6.01. A *p*-value <0.05 was considered statistically significant.

## Results

### Effect of FJG and met on body weight, FBG, insulin level, lipid profile, and renal function

After six weeks of the high-fat high-sucrose diet and treatments, the ZDF (fa/fa) rats in vehicle-treated DM group displayed typical diabetic symptoms and body weight gain, along with dull fur and a reduced activity level, compared to the normal controls. Rats in the Met, FJG, and Met + FJG groups exhibited improved clinical symptoms, compared with rats in the DM group.

**Figure 1.**
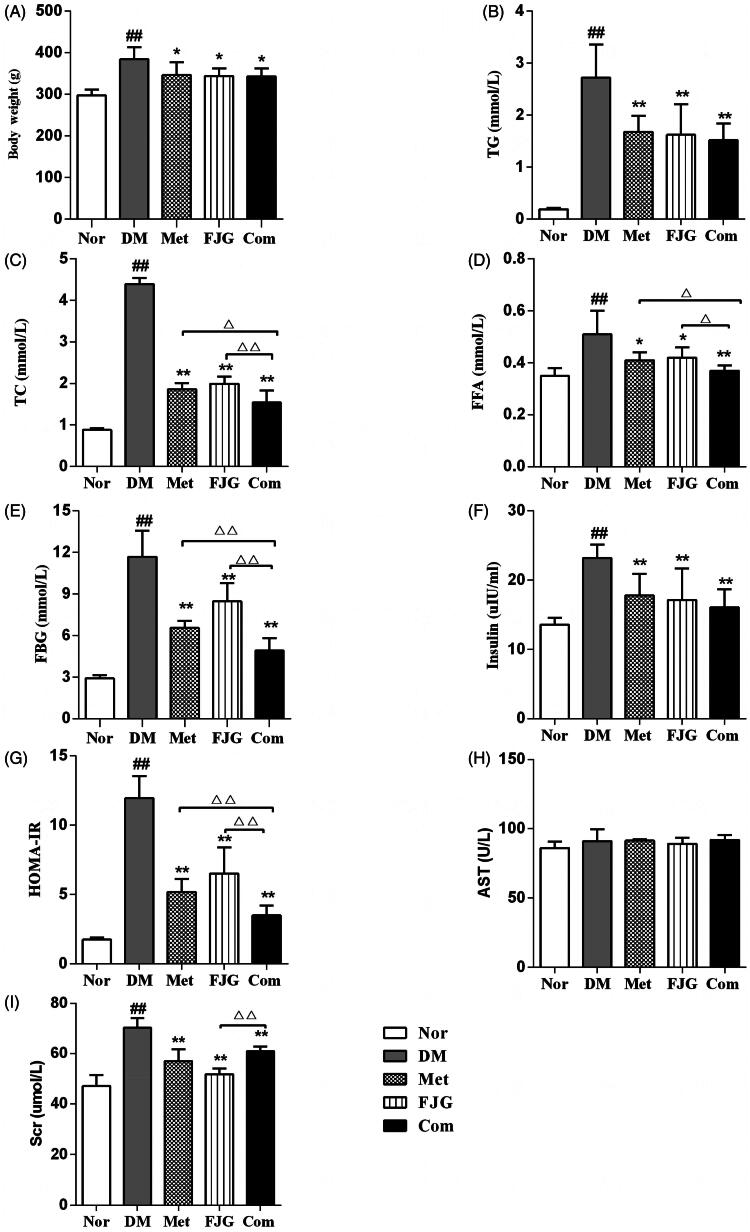
Met and FJG treatment ameliorated diabetes in ZDF rats. (A) FBG, (B) insulin, (C) HOMA-IR, (D) body weight, (E) TG, (F) TC (G) FFA (H) AST (I) SCr of ZDF rats (*n* = 6 each group) were measured. #*p* < 0.05, ##*p* < 0.01 as compared to the Nor group; *p < 0.05, **p < 0.01 as compared to the DM group; ^△^*p* < 0.05, ^△△^*p* < 0.01 as compared to the Met + FJG group.

The rats in the DM group were significantly overweight (384.50 ± 29.37 g) compared to the rats in the normal group (297.67 ± 14.15 g, *p* < 0.01). However, the body weight of rats was significantly decreased following the six-week treatment with Met (346.50 ± 30.61 g), FJG (344.17 ± 18.32 g) and Met + FJG (343.17 ± 19.68 g) as compared to the DM group (*p* < 0.05; Figure 1), although they still weighed more than normal controls (*p* < 0.05 for all).

Compared with the normal control, the rats in the DM group had significantly higher levels of FBG, FINS, HOMA-IR, TG, TC, FFA and SCr (*p* < 0.01, Figure 1). However, treatment with either FJG or Met significantly decreased the levels of these indicators (*p* < 0.05 or <0.01). Interestingly, the Met + FJG group achieved the maximum reduction in FBG, HOMA-IR, TC, and FFA compared to the DM group, suggesting a synergistic therapeutic effect of Met and FJG on diabetes.

### Effect of FJG and met on glucose tolerance in diabetic rats

**Figure 2.**
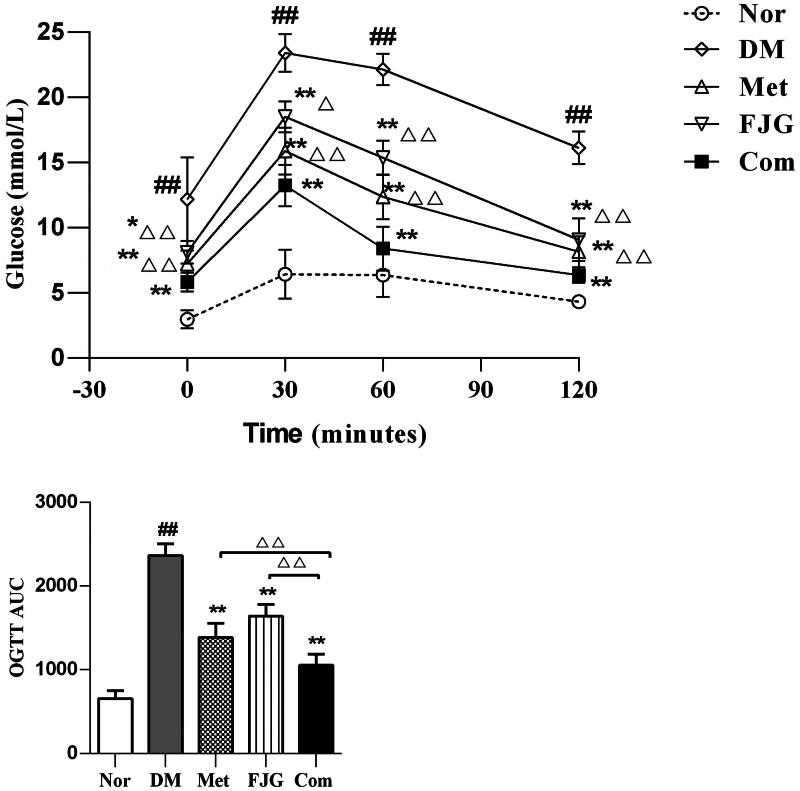
Met and FJG treatment improved glucose tolerance in ZDF rats. Blood glucose level and AUC of OGTT tests in ZDF rats (*n* = 6 each group) were measured. #*p* < 0.05, ##*p* < 0.01 as compared to the Nor group; *p < 0.05, **p < 0.01 as compared to the DM group; ^△^*p* < 0.05, ^△△^*p* < 0.01 as compared to the Met + FJG group.

The rats in the DM group had significantly higher glucose concentrations at all time points during the OGTT, as well as a greater AUC when compared to the normal controls (*p* < 0.001). However, both Met and FJG individually decreased the glucose levels at all time points, and reduced the AUC when compared to the DM group (*p* < 0.05 or <0.01). In addition, treatment with Met + FJG yielded the greatest hypoglycemic outcomes as well as smallest AUC (Figure 2).

### Antioxidant potential of FJG and met in ZDF rats

**Figure 3.**
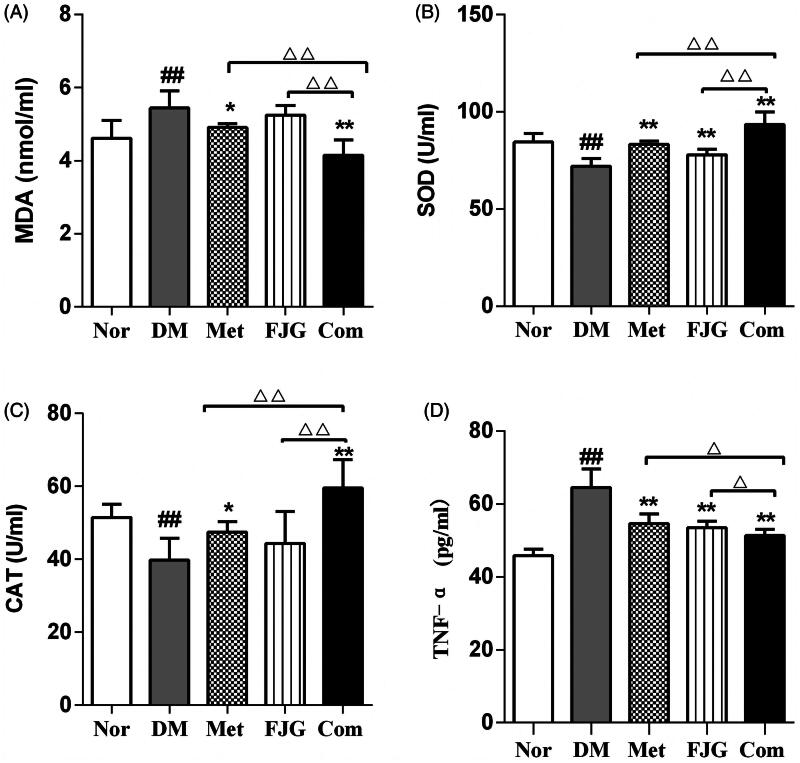
Met and FJG treatment ameliorated oxidation in ZDF rats. (A) MDA, (B) SOD, (C) CAT, (D) TNF-α levels in ZDF rats (*n* = 6 each group) were measured. #*p* < 0.05, ##*p* < 0.01 as compared to the Nor group; *p < 0.05, **p < 0.01 as compared to the DM group; ^△^*p* < 0.05, ^△△^*p* < 0.01 as compared to the Met + FJG group.

The rats in the DM group exhibited increased levels of MDA and TNF-α, and decreased SOD and CAT activities, when compared to the normal controls (*p* < 0.01, Figure 3). Met treatment effectively reduced MDA production, decreased the level of TNF-α, and increased SOD and CAT activities when compared to the DM group (*p* < 0.05 or <0.01). Similarly, treatment with FJG also resulted in a decreased level of in TNF-α and an increase in SOD activity, as compared to the DM group (*p* < 0.01). Taken together, these data suggest that FJG could improve the effect of Met by also reducing oxidative stress in diabetic rats.

### Histological examination

**Figure 4.**
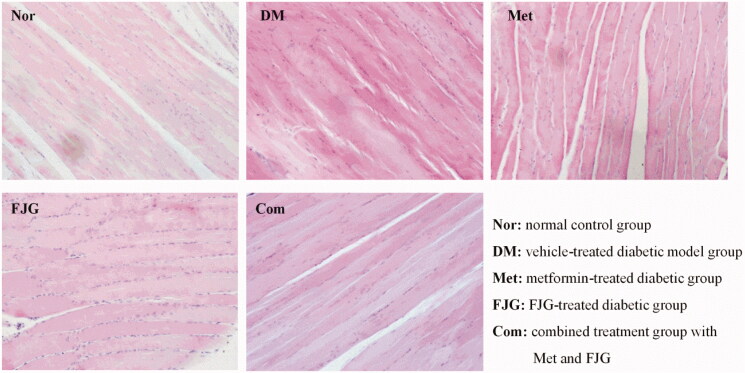
Histological findings in skeletal muscle. Both Met and FJG, particularly in combination, remarkably restored histological changes of damaged skeletal muscle fibers. Images are representative of *n* = 6 in each group. Magnification: 200×.

In H&E stained sections, the normal group exhibited normal morphology of skeletal muscle fibers, which were neatly arranged with homogeneous cytoplasm and regular nuclei. In contrast, the skeletal muscle fibers of rats in the DM group were disordered, with non-homogenous cytoplasm and the appearance of a nuclear shift. A small amount of inflammatory cell infiltration was also observed. Met and FJG, particularly in combination, remarkably restored the histological changes of damaged skeletal muscle fibers (Figure 4).

### FJG and met regulated the mRNA expression of insulin-signaling pathway components in the skeletal muscle of ZDF rats

**Figure 5.**
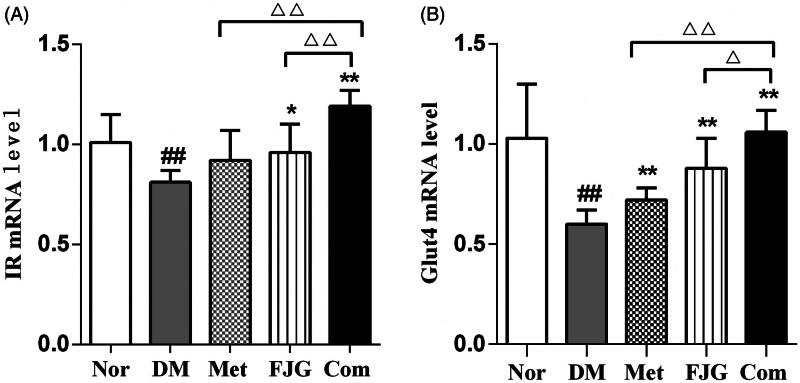
Met and FJG treatment regulated the mRNA expression of InsR and Glut4 in diabetic ZDF rats. (A) InsR (B) Glut4 in skeletal muscle of ZDF rats (*n* = 6 each group) were measured. #*p* < 0.05, ##*p* < 0.01 as compared to the Nor group; *p < 0.05, **p < 0.01 as compared to the DM group; ^△^*p* < 0.05, ^△△^*p* < 0.01 as compared to the Met + FJG group.

Compared with normal control rats, the rats in the DM group had significantly decreased mRNA levels of InsR and Glut4 (*p* < 0.01, Figure 5) in skeletal muscle tissue. Met administration resulted in a significant increase in the mRNA level of Glut4 compared to the DM group (*p* < 0.01). Similarly, treatment with FJG alone significantly increased mRNA levels of InsR and Glut4, as compared to the DM group (*p* < 0.05 or <0.01). Met and FJG in combination up-regulated the mRNA expression of both InsR and Glut4, as compared to Met or FJG alone (*p* < 0.05 or *p* < 0.01).

### FJG and met regulated the protein expression of insulin-signaling pathway components in the skeletal muscle of ZDF rats

Compared with normal control rats, the protein levels of InsR, p-IRS-1 tyr989, m-PI3Kp85, p-Akt and Glut4 were significantly down-regulated in skeletal muscle tissue of rats in the DM group, whereas p-IRS-1 (ser1101, ser612, ser307) was significantly up-regulated (*p* < 0.05 or <0.01; Figure 6). To varying degrees, treatment with Met or FJG restored the abnormal expression or phosphorylation status of these proteins. Importantly, Met and FJG in combination achieved the greatest potential for reversing the decreased levels of InsR, p-IRS1 tyr989, m-PI3Kp85, p-Akt, and Glut4, and elevated levels of p-IRS-1 (ser1101, ser612, ser307), as compared to Met or FJG alone (*p* < 0.05 or <0.01).

Figure 6.Met and FJG treatment regulated protein levels of components in the insulin-signaling pathway in skeletal muscle of ZDF rats. The protein levels of InsR (A), p-IRS-1 ser1101(B), p-IRS-1 ser612 (C), p-IRS-1 ser307 (D), p-IRS-1 tyr989 (E), m-PI3Kp85 (F), p-Akt (G), Glut4 (H) were determined by western blot analysis. #*p* < 0.05, ##*p* < 0.01 as compared to the Nor group; *p < 0.05, **p < 0.01 as compared to the DM group; ^△^*p* < 0.05, ^△△^*p* < 0.01 as compared to the CT group.

**Figure:**
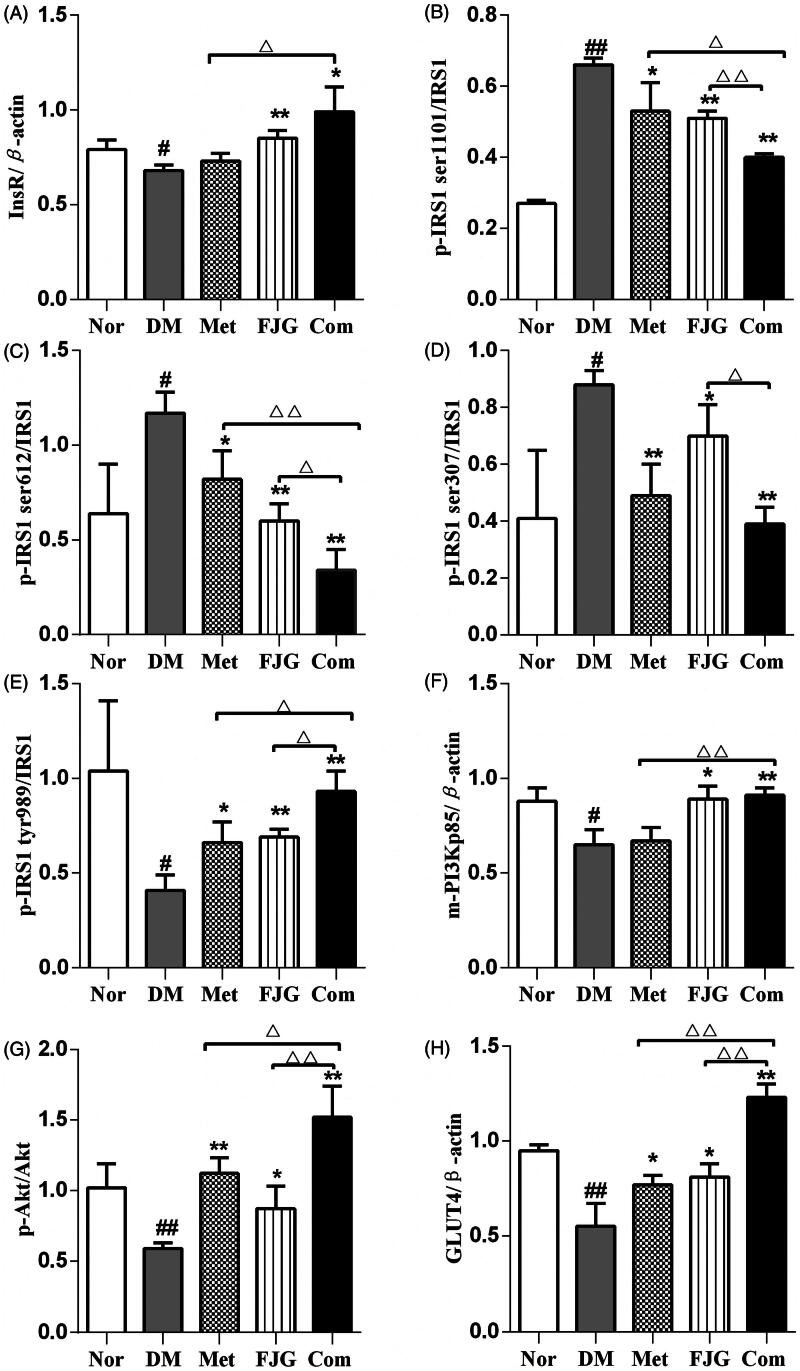

**Figure:**
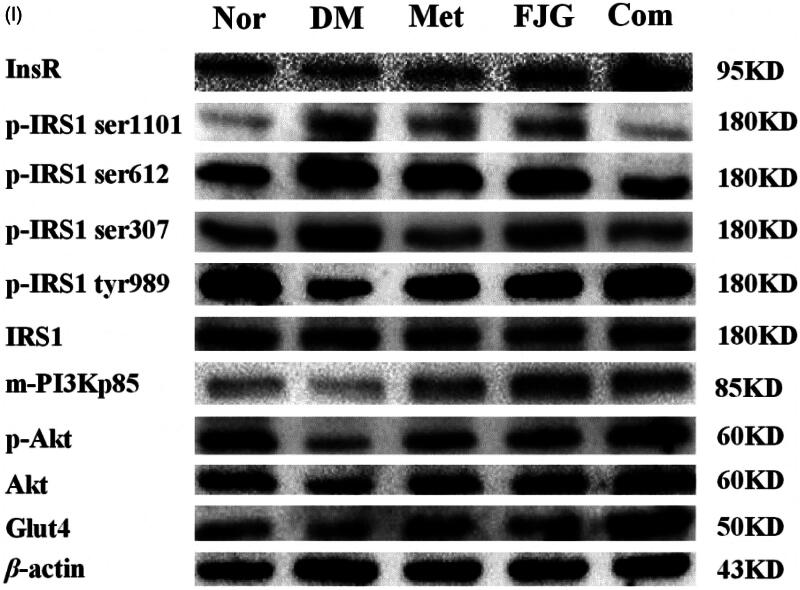

## Discussion

The ZDF (fa/fa) rat is a widely used and important model for type 2 diabetes since it exhibits impaired glucose tolerance with the inherited insulin-resistance gene (Yokoi et al. 2013). This model closely simulates diabetic manifestations, including obesity, hyperglycemia and insulin resistance, as well as hyperlipidemia and renal dysfunction. In this study, treatment with FJG and Met for six weeks remarkably decreased the levels of FBG and insulin. Interestingly, FJG improved the Met-mediated effect of lowering FBG and insulin resistance, as evidenced by the reduced AUC in OGTT tests and decreased HOMA-IR index. Consistently, FJG partially restored histological abnormalities of skeletal muscle in ZDF (fa/fa) rats. It was also observed that the concomitant use of Met and FJG achieved a synergistic therapeutic effect against diabetes.

Obesity and dysregulated lipid metabolism are important contributing factors to insulin resistance (Savage et al. 2007; Jung and Choi 2014). We observed that oral administration of Met and FJG restored the pathological changes of TC, TG and FFA in ZDF (fa/fa) rats. The effect of FJG on modifying lipid profiles might contribute to the regulation of insulin resistance. In addition, FJG-mediated decreased bodyweight in ZDF (fa/fa) rats, which relates to decreased blood glucose levels, alleviated insulin resistance and reduction of lipid formation. Also, FJG effectively reversed the elevation of SCr in ZDF (fa/fa) rats to a level even superior to that of the Met group, revealing a potential renoprotective effect of FJG.

Diabetes is accompanied by enhanced production of free radicals and diminished antioxidants including SOD, CAT and glutathione peroxidase (GSHPx). Previous evidence has revealed hyperlipidemia-induced ROS production and an accumulation of damaged mitochondria in skeletal muscle (Di Meo et al. 2017). MDA, as one of the end products of membrane lipid peroxidation, has widely served as a sensitive marker of oxidative stress. TNF-α, one of main cytokines released in the diabetic inflammatory processes, mediates its effects by binding to its receptors, TNF-R1 and TNF-R2 (Rahal et al. 2014). A previous study has demonstrated that decreased TNF-α mediated by *Ixeris gracilis* Stebb. (Asteraceae) [syn. *Lactuca gracilis* (Wall.) DC.] contributes to the antioxidative and antidiabetic potential of plants extracts (Syiem and Warjri 2015). In this study, compared with vehicle-treated ZDF (fa/fa) rats, FJG treatment resulted in the recovery of the decreased levels of SOD and CAT activity accompanied with the inhibition of MDA. Moreover, FJG reduced the elevated level of TNF-α in diabetic rats to a level comparable with the Met group. A combination of Met and FJG displayed a synergistic effect on reducing oxidative stress in diabetic rats. These data suggested that FJG, alone and in combination with Met, could remove toxic free radicals and therefore protect skeletal muscle against cellular injury caused by oxidative stress.

The PI3K/Akt signaling pathway is involved in glucose metabolism and insulin-related signal transduction. After the binding of insulin to its receptors on the membrane of target cells, insulin modulates the serine or/and tyrosine phosphorylation of IRS-1 and the subsequent activation of PI3K and Akt by phosphorylation, which in turn regulates glucose and lipid metabolism (Zdychová and Komers 2005; Leto and Saltiel 2012). The activation of PI3K, as an intermediate in the insulin signaling pathway, leads to Glut4 translocation to the membrane and enhanced glucose transport (Huang and Czech 2007; Leto and Saltiel 2012). The insulin-stimulated PI3K/Akt activity has been reported to be lower in skeletal muscle of patients with type 2 diabetes (Kim et al. 2003; Jensen et al. 2008). A previous study has also shown down-regulation of PI3K signaling in the skeletal muscle of type 2 diabetic KKAy mice (Zhang et al. 2016). Met-mediated decreased glucose has mainly been attributed to its ability to increase peripheral insulin sensitivity and/or promote insulin-mediated glucose uptake in the skeletal muscle of type 2 diabetic patients. These effects are in part mediated by stimulating Glut4 translocation and promoting insulin to bind to its receptors on the cell surface (Choi and Kim 2010). In this study, FJG, alone or in combination with Met, was found to significantly increase the suppressed mRNA and protein expression of IRS-1 and Glut4 in diabetic rats. By western blot analysis, phosphorylation of IRS-1 was significantly increased at serine sites (ser-110, -612 and -307) and decreased at a tyrosine site (tyr-989) in vehicle-treated ZDF (fa/fa) rats. Met or FJG treatment, especially in combination, effectively reversed the abnormal expression of these proteins. In addition, FJG partially corrected the abnormal protein levels of IRS-1, PI3K, Akt and Glut4 in ZDF (fa/fa) diabetic rats, suggesting a regulatory effect of FJG by alleviating insulin resistance in skeletal muscle through modulation of the PI3K signaling pathway.

## Conclusions

This study shows that FJG, especially in combination with Met, has beneficial effects on lowing FBG, alleviating insulin resistance, modulating lipid profiles, reducing SCr levels, as well as partially restoring glucose tolerance. The effect of FJG on alleviating the metabolic symptoms of diabetic rats is likely through improved antioxidant activity, as evidenced by reducing MDA and TNF-α levels, increasing SOD and CAT activity, and regulating the PI3K/Akt signaling pathway in skeletal muscle. Our study has provided a theoretical basis for FJG, alone or in combination with Met, to improve insulin resistance in diabetic patients.
